# Recombinant expression, characterization, and quantification in human cancer cell lines of the Anaplastic Large-Cell Lymphoma-characteristic NPM-ALK fusion protein

**DOI:** 10.1038/s41598-020-61936-w

**Published:** 2020-03-19

**Authors:** Katerina Kourentzi, Mary Crum, Ujwal Patil, Ana Prebisch, Dimple Chavan, Binh Vu, Zihua Zeng, Dmitri Litvinov, Youli Zu, Richard C. Willson

**Affiliations:** 1 Department of Chemical & Biomolecular Engineering, University of Houston, Houston, TX 77204 USA; 2 Department of Biology & Biochemistry, University of Houston, Houston, TX 77204 USA; 3 Escuela de Medicina y Ciencias de la Salud ITESM, Monterrey, Mexico; 4 Department of Pathology and Genomic Medicine, Houston Methodist Hospital, Houston, TX USA; 5 Department of Electrical & Computer Engineering, University of Houston, Houston, TX USA

**Keywords:** Assay systems, Haematological cancer

## Abstract

Systemic anaplastic large cell lymphoma (ALCL) is an aggressive T-cell lymphoma most commonly seen in children and young adults. The majority of pediatric ALCLs are associated with the t(2;5)(p23;q35) translocation which fuses the Anaplastic Lymphoma Kinase (ALK) gene with the Nucleophosmin (NPM) gene. The NPM-ALK fusion protein is a constitutively-active tyrosine kinase, and plays a major role in tumor pathogenesis. In an effort to advance novel diagnostic approaches and the understanding of the function of this fusion protein in cancer cells, we expressed in *E. coli,* purified and characterized human NPM-ALK fusion protein to be used as a standard for estimating expression levels in cultured human ALCL cells, a key tool in ALCL pathobiology research. We estimated that NPM-ALK fusion protein is expressed at substantial levels in both Karpas 299 and SU-DHL-1 cells (*ca*. 4–6 million molecules or 0.5–0.7 pg protein per cell; based on our *in-house* developed NPM-ALK ELISA; LOD of 40 pM) as compared to the ubiquitous β-actin protein (*ca*. 64 million molecules or 4.5 pg per lymphocyte). We also compared NPM-ALK/ β-actin ratios determined by ELISA to those independently determined by two-dimensional electrophoresis and showed that the two methods are in good agreement.

## Introduction

**Figure 1:**
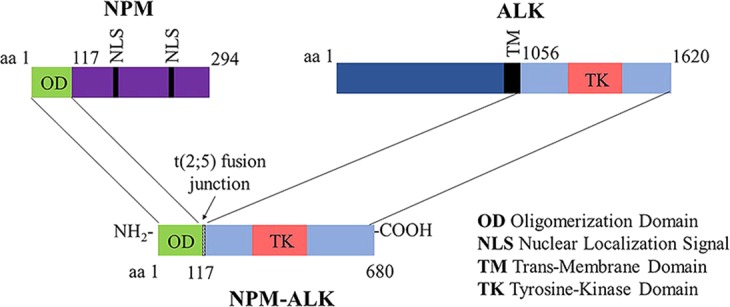
The chromosomal translocation t(2;5)(p23;q35) results in production of the NPM-ALK fusion gene and NPM-ALK fusion protein. The Oligomerization Domain (OD) of NPM provides the biochemical basis for NPM-ALK fusion protein oligomerization, allowing auto-phosphorylation and constitutive activation of NPM-ALK that plays a major role in the pathogenesis of ALK^+^ ALCL.

Systemic anaplastic large cell lymphoma (ALCL) is an aggressive CD30^+^ T-cell lymphoma most commonly seen in children and young adults under 30 years of age (with a moderate male prevalence), and comprises approximately 10–20% of pediatric lymphomas. Almost all pediatric ALCLs express oncogenic anaplastic lymphoma kinase (ALK) fusion proteins due to a translocation of the ALK gene locus on chromosome 2p23. In almost 90% of ALK-positive (ALK^+^) ALCL, the translocation fuses the ALK kinase gene to the nucleophosmin (NPM) gene at chromosome locus 5q35 and results in the expression of an oncogenic fusion protein, NPM-ALK (Fig. 1)^1–4^. In the remaining patients, the ALK gene is fused to TPM3, TFG, ATIC and other rarer genes^5,6^. The NPM-ALK fusion oncogene protein (680 amino acids; MW: 75 kDa) consists of the 563 C-terminal amino acids of anaplastic lymphoma kinase (starting at amino acid 1058; the entire intracytoplasmic region) fused to the first 117 N-terminal amino acids of the ubiquitously-expressed nucleolar phosphoprotein nucleophosmin (NPM)^1^. NPM (MW: 23 kDa) is a molecular chaperone that shuttles between the nucleus and cytoplasm and is involved in regulating cell division and DNA repair and maintaining genomic stability^7^. ALK (MW: 180 kDa; MW about 220 kDa after N-linked glycosylation of 16 sites, mostly asparagines^8^), is a tyrosine kinase of the insulin receptor tyrosine kinase superfamily, whose normal function is not well understood. ALK mRNA was initially identified in t(2;5)-positive cell lines and patients by cDNA Southern blot and RNA Northern blot analyses by Morris *et al*.^1^. Full length ALK mRNA was also detected in the rhabdomyosarcoma-derived cell line Rh30 (and later in a broad range of ectodermal cell lines^9^) whereas ALK transcripts were not detected in hematopoietic cells including peripheral blood leukocytes^1^. Later studies showed that the full-length ALK protein is highly expressed in the neonatal murine brain but its level falls by 3 weeks after birth^10,11^. Pulford *et al*. showed that ALK protein in normal tissues is restricted to the brain (in scattered neurons, glial cells, and endothelial cells), suggesting that ALK protein is involved in neuronal development and differentiation^12^. When fused to ALK, NPM contributes an active promoter which drives expression of the ALK catalytic domain, and constitutive activation of ALK contributes to tumorigenesis by deregulating a number of signal transduction pathways, including those of JAK/STAT3, PI3K/AKT, Ras/MAPK, mTOR, MEK/ERK, and JNK/c-Jun, increasing cell proliferation and resistance to apoptosis^13,14^. Several other ALK fusion proteins also have been identified in various solid tumors^15^, most notably in neuroblastomas and non-small cell lung cancers^16^.

Since the detection of ALK fusion protein in lymphocytes correlates perfectly with the presence of the ALK chromosomal fusion, immunohistochemistry (IHC) with specific anti-ALK monoclonal antibodies^4,17^ has displaced DNA molecular tests for the diagnosis of ALK-positive ALCL (FISH detection of ALK gene translocations is technically possible but labor- and time-consuming, and costly). ALCL cells are large and heterogeneous (the prognostic impact of the morphologic variants has been reported^18,19^) with abundant cytoplasm and eccentric, lobulated nuclei. In most cases with NPM-ALK fusion, ALK staining is both cytoplasmic and nuclear. In cases with other, non-NPM fusions, ALK staining is usually cytoplasmic. NPM-stained cells with NPM-ALK fusion show aberrant cytoplasmic expression of NPM, whereas cases with other non-NPM fusions show the expected nuclear-restricted expression of NPM. ALCL cells also display variable loss of CD3 and other T-cell markers, but strong expression of CD30. Although technically well-established and revealing cell morphology that may correlate with prognosis^18^, IHC is labor- and skill- intensive, absolute quantification is challenging, and an adequate amount of tissue is not always available for analysis^20,21^. Messenger RNA level is used as an indirect measure of protein expression^22^ and thus detection of NPM-ALK mRNA with RT-PCR^19,23^ might be an alternative approach for ALCL diagnosis, but mRNA instability and susceptibility to degradation largely limit its use as a reliable biomarker for ALCL diagnosis. Precise protein quantification is useful in overall disease detection, therapy monitoring, minimal residual disease detection and early detection of relapse. Aberrant phosphorylation by NPM-ALK fusion protein contributes directly to ALCL pathogenesis, and high expression of the NPM-ALK fusion mRNA has been associated with a poorer prognosis^23^ (though ALK-positive ALCL is generally associated with a favorable clinical outcome).

While mass spectrometry-based methods^24–27^ are advancing, clinical protein quantitation most commonly relies on antibody-based approaches, with ELISA the current standard for affinity-based quantitative protein measurements. Flow cytometry immunophenotyping is useful as a complementary technique but the definite diagnosis of ALCL by flow cytometry remains challenging due to the low occurrence, fragility, and size- and shape-variability of ALCL cells^5,28^.

Aside from the clinic, the quantification of the level of the fusion protein in the commonly used ALCL human cell lines^29^ has not been reported but is of the greatest interest. ALCL human cell lines are fundamental cancer research tools which provide a pure, stable and consistent population of cells. ALK^+^ Karpas 299 and SU-DHL-1 ALCL cell lines have been instrumental in the cloning of the genes involved in t(2;5) translocation, have served as references for studies on biopsy material^29,30^, have been used to elucidate the molecular mechanisms of these specific malignancies^27,31–34^ and are ultimately useful for drug development and preclinical testing^31,35–37^.

Motivated by the facts that expression of full-length ALK is absent in hematopoietic cells, and that NPM-ALK fusion protein is a specific biomarker of ALCL with a long half-life of 48 hours^38^ we developed an immunoassay for NPM–ALK fusion protein by using human ALK^+^ ALCL cell lines after screening various antibodies that recognize both the ALK and the fusion portion of the NPM-ALK fusion protein. Since no source of the fusion protein is available, we cloned, expressed in *E. coli*, purified and characterized recombinant NPM-ALK fusion protein to be used as a positive control and calibration standard. We then used it to determine NPM-ALK fusion protein expression levels in ALCL cell lines and to confirm the NPM-ALK/ β-actin ratios determined by ELISA using those independently determined by two-dimensional electrophoresis and we estimated that NPM-ALK fusion protein is expressed at substantial levels in human ALCL as compared to the ubiquitous β-actin protein.

## Results and Discussion

### Production and purification of recombinant NPM-ALK fusion protein

The human NPM-ALK gene was cloned into a pET28a plasmid vector, and overexpression of the recombinant his_6_-tagged NPM-ALK fusion protein in *E. coli* resulted in the formation of inclusion bodies. Initial trials of on-column refolding^39^ on Ni-NTA Sepharose column led to excessive visible aggregation and were deemed unsuccessful. Protein refolding was achieved by slow, dropwise, dilution of urea-solubilized inclusion bodies into a larger volume of refolding buffer containing 146 mM sucrose and 400 mM L-arginine, two commonly-used aggregation suppressors^40^. After refolding, histidine affinity tag-directed metal chelate affinity chromatography initially was utilized to purify the refolded NPM-ALK fusion protein. To further discriminate against possible misfolded variants of the protein (whose presence was suggested by differing A280-normalised ELISA signals among nearly-electrophoretically-pure IMAC column fractions) anion-exchange chromatography was used to eliminate any conformational variants. The recombinant protein was eluted over 8 ml in a single peak at 350 mM NaCl (Fig. S1). Two 1-ml fractions were collected (at 5–6 and 6–7 ml), pooled, exchanged in 25 mM Tris-HCl, aliquoted and stored at −20 °C in the presence of 50% glycerol. The absence of protein in the flow-through indicated the presence of largely anionic protein species binding to the Q-Sepharose at pH 8.0.

### Characterization of recombinant NPM-ALK fusion protein

The theoretical protein MW was estimated at 75,314.22 Da using the ExPASy Compute pI/MW tool and confirmed by SDS-PAGE, and protein concentration was quantified by BCA protein assay. The sequence of the purified recombinant NPM-ALK protein was characterized with tryptic peptide LC-MS/MS mapping at the Proteomics Facility, U.T. MD Anderson Cancer Center, Houston, TX. The mapping yielded 59.4% coverage of the total sequence of NPM-ALK by the identified peptides (Fig. S2).

### Cell lysis optimization

**Figure 2:**
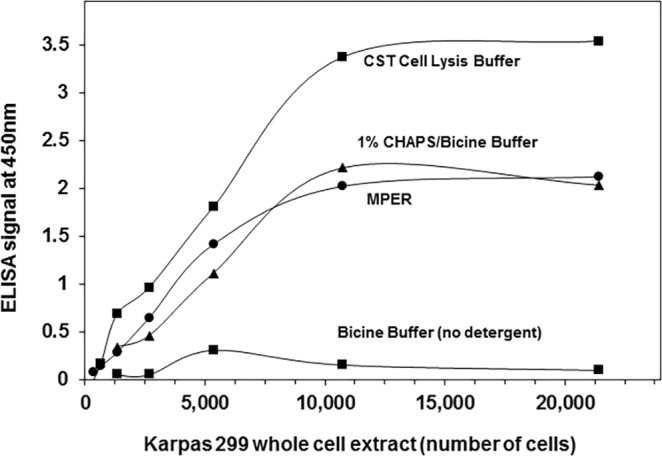
Effect of different lysis reagents on NPM-ALK protein ELISA detection in whole cell extracts. NPM-ALK-expressing Karpas 299 lymphoma cells were lysed with various lysis reagents. Serial dilutions of the cell extracts were tested for the released NPM-ALK protein using the PathScan Total ALK ELISA kit (CST #7322). Total protein content in the whole cell lysates was measured by the bicinchoninic acid (BCA) method against a BSA standard curve and was estimated to be in the range of 100–160 pg of protein per cell.

Efficient and non-denaturing extraction of intracellular proteins from cells is essential for downstream immunoassays. Complete cell lysis with a mild detergent is commonly used, as low detergent concentrations (e.g. 1% Triton X-100) are sufficient to disrupt cell membranes to liberate total protein from most cellular compartments^41,42^. Extracts of NPM-ALK-expressing Karpas 299 cells were prepared with different non-denaturing lysis reagents and tested by ELISA for immunodetection of NPM-ALK fusion protein. The Cell Lysis Buffer from Cell Signaling Technology gave the best performance (the highest 450 nm absorbance in the presence of cell lysate) of the downstream ELISA, better than M-PER (containing the zwitterionic detergent CHAPS in buffered Bicine solution^43^) and an *in-house*-formulated solution (1% CHAPS in 25 mM Bicine Buffer, pH 7.5) (Fig. 2). Only background signal was observed when cells were resuspended in Bicine Buffer in the absence of any detergent, confirming the need for a detergent-based cell lysis. As shown below, cells not expressing the NPM-ALK fusion protein but lysed with the Cell Lysis Buffer gave only background ELISA signal confirming the specificity of the NPM-ALK ELISA signal with the positive Karpas 299 cells.

### Antibody screening

Several monoclonal antibodies were evaluated in pairwise combinations^44,45^ in a sandwich ELISA both for capture and detection. We chose to test the antibodies in their native form (combined with a secondary anti-species HRP-conjugated antibody) instead of their biotinylated form to avoid altering their binding properties. This design limited the total number of combinations to be tested since only antibodies coming from different species could be combined in a specific pair, but allowed for reliable and efficient screening. Based on the manufacturers’ specifications, we tested antibodies that recognize the NPM or the ALK parts of the fusion protein, and antibodies raised against synthetic fusion peptides. Since NPM is a ubiquitously, abundantly expressed protein, antibodies that recognize the NPM protein were deemed unsuitable for the specific capture of the fusion protein and were tested only as detection antibodies. On the contrary, since ALK expression is restricted to the central and peripheral nervous system, with minimal or no expression in other normal tissues, anti-ALK antibodies were tested for both capture and detection of the fusion protein with an *a priori* preference for their use in detection, as described below. Whole-cell extracts from 5,000 Karpas 299 cells were used as the positive control. Jurkat cells (20,000 cells; negative for the fusion protein) were used to assess the extent of non-specific binding (Fig. S3). Antibody pairs that included anti-ALK antibody #3791 as the detection antibody (a mouse monoclonal IgG targeting an ALK C-terminus fragment included in the NPM-ALK fusion protein) produced the highest specific signal and thus anti-ALK antibody #3791 was chosen as the detection antibody. Of all the antibody pairs tested, the #3333 (capture) /#3791 (detection) antibody pair had the highest specific signal; this antibody pair is used in a commercial PathScan total ALK ELISA kit (Cell Signaling Technology). Interestingly, the same pair showed a 65% decrease in signal when capture/detection role of antibodies was reversed. Of the two antibodies that recognize the fusion protein, only ab180607 (a recombinant rabbit monoclonal antibody raised against a short peptide around the fusion junction of NPM-ALK fusion protein) gave a satisfactory performance when used as a capture antibody. The performance of the ab180607/#3791 pair was not affected by inverting antibody roles in capture or detection. Given that our goal was to develop a NPM-ALK fusion protein-specific assay and not an ALK-specific assay, we chose ab180607 against the junction of the fusion NPM-ALK protein as the capture antibody.

### ELISA characterization with recombinant standard NPM-ALK

**Figure 3:**
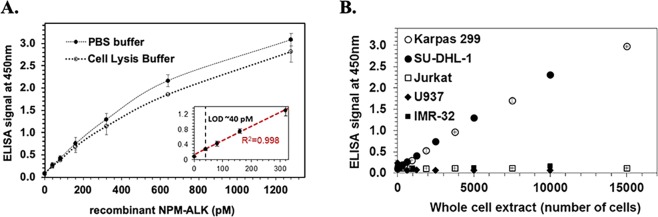
(**A**) ELISA characterization with recombinant NPM-ALK fusion protein. Two-fold serial dilutions of NPM-ALK recombinant protein were spiked in PBS buffer or Cell Lysis Reagent and used in the ELISA with the ab180607 (capture) /#3791 (detection) antibody pair (n = 3, average ± 1 SD). An HRP-conjugated horse anti-mouse IgG antibody (#7076) from Cell Signaling Technology along with 1-Step™ Ultra TMB-ELISA Substrate Solution was used for signal development. (Inset) The fitted 5-point linear calibration curve is shown with the red dotted line; estimated LOD is shown with the black dotted line. (**B**) **NPM-ALK ELISA specificity**. Immunodetection of cellular NPM-ALK protein in cultured human lymphoma (Karpas 299, SU-DHL-1, Jurkat, U937) and neuroblastoma (IMR-32) cells by ELISA with the ab180607 (capture) /#3791 (detection) antibody pair (n = 3, average ± 1 SD). Whole cell extracts were prepared in Cell Lysis Buffer. An HRP-conjugated horse anti-mouse IgG antibody (#7076) from Cell Signaling Technology along with 1-Step™ Ultra TMB-ELISA Substrate Solution was used for signal development.

As shown in Fig. 3A, we confirmed the picomolar detection of the recombinant NPM-ALK protein with the ab180607 (capture)/#3791 (detection) antibody pair both in PBS and in cell lysis buffer (confirming that the presence of the lysis reagent used for the treatment of the cancer cell lines does not affect the performance of the immunoassay). Signal linearity was observed in the picomolar range (0–320 pM). A calibration curve was constructed by fitting a straight line through the linear portion of the data (0–320 pM). The Limit of Detection (LOD), estimated as the lowest concentration with average signal above the mean plus three standard deviations of the no-target negative control, was 40 pM (3.2 ng/mL). Spiking the recombinant NPM-ALK protein in negative Jurkat cell lysate (~30,000 cells) showed > 90% signal recovery. We also confirmed that the recombinant NPM-ALK protein is recognized by the commercial total ALK #3333 (capture)/#3791 (detection) antibody pair with similar sensitivity.

### Quantitation of cellular NPM-ALK

Whole cell lysates from the NPM-ALK positive ALCL cell lines Karpas 299 and SUDHL-1 were 2-fold serially diluted in PBS. The number of cells had been determined previously by cell counting. Signal linearity was observed in the range of 300–10,000 cells (Fig. 3B). No significant signal was observed with the U937 or Jurkat cell lysates (even at 60,000 cells) that are negative for both ALK and NPM-ALK-fusion protein. Most importantly, no significant signal was observed with the IMR-32 neuroblastoma cell line that expresses the full-length wild-type ALK protein^9,46^ but not the ALCL-specific NPM-ALK fusion protein. When NPM-ALK-positive cell lysate was spiked into negative pooled human serum (purchased from the Gulf Coast Regional Blood Center, Houston, TX), the slope of the signal versus the number of cells decreased as the concentration of serum increased but even at 50% serum the assay still provided a similar quantification range as when cells were spiked in PBS/1% BSA buffer. However, cells will be ultimately isolated from whole blood (or buffy coat) and washed prior to the immunoassay and thus minimizing the presence of serum or other blood components. Mixing experiments were performed to estimate the sensitivity of the NPM-ALK ELISA to detect ALK-positive ALCL cells in whole cell lysates. Karpas 299 cells were serially diluted in 2 million ALK-negative U937^47^ cells and whole cell extracts were prepared in Cell Lysis Buffer. We observed a sensitivity of 5 × 10^−3^ (0.5% Karpas 299 ALCL cells; Fig. 5S).

Using the recombinant NPM-ALK fusion protein as the calibration standard and a 5-point ELISA calibration curve (Fig. 3A) performed in the same 96-well plate, we estimated the expression level of the NPM-ALK fusion protein in the cultured Karpas 299 human ALCL cells at ca. 4–6 million copies (0.5–0.7 pg) of protein per cultured cell.

A sensitive immunoassay is a useful tool for NPM-ALK specific protein quantification and may be widely useful in cancer biology studies. We are separately working on adapting our immunoassay to magnetic-based detection with greatly enhanced detection sensitivity and matrix-insensitivity. Based on preliminary experiments, we believe there is a realistic possibility we will be able to detect NPM-ALK fusion protein in buffy coat lysates from peripheral blood samples, instead of (invasive) tissue biopsies using our (under-development) magnetic immunosensors^48,49^ and/or other methods. Thus we believe that an ultrasensitive immunoassay coupled to a matrix-insensitive magnetic reader may serve as a complementary diagnostic test to current procedures and also be used for minimal residual disease detection, recurrence, and treatment efficacy monitoring in patients harboring the NPM-ALK fusion. Given that around 10% of ALK^+^ ALCL cases harbor a different ALK-fusion protein, we note that the same methodology can be used for development of an immunoassay targeting the other, rarer ALK-fusion proteins.

### Immunofluorescence - based measurement of NPM-ALK expression in ALCL cells

For independent validation of the ELISA results, the expression level of NPM-ALK protein in Karpas 299 ALCL cells was determined by fluorescently-stained two-dimensional gel electrophoresis (Fig. S4). Densitometric analysis of the gel reveals that the expression level of NPM-ALK is high, at a relative molar ratio of cellular β-actin (one of most abundant cellular proteins) to NPM-ALK protein approximately at 80–100:1. Assuming the average actin content per lymphocyte is *ca*. 4.5 pg^50^ (or 64 million molecules) we estimate *ca*. 0.8–1.1 million molecules of NPM-ALK fusion protein per cell consistent with our ELISA results of *ca*. 4–6 million copies (0.5–0.7 pg) of protein per cell.

In conclusion, we developed and validated a sensitive immunoassay for the quantification of NPM-ALK protein using recombinant and cellular proteins derived from cultured human lymphoma cells. Recombinant NPM-ALK protein to be used as a positive control and calibration standard, was produced, refolded, purified and characterized in-house. This study represents the first availability from any source of this important oncoprotein, which may be widely useful in cancer biology beyond the present work. Furthermore, we confirmed that there is a substantial NPM-ALK fusion protein expression in ALCL cells, measured for first time to be ca. 4–6 million copies (0.5–0.7 pg) of protein per cell, and independently confirmed by two-dimensional electrophoresis.

## Methods

### Production of recombinant fusion protein

The human NPM-ALK gene in the pCDNA3/NPM-ALK construct was kindly provided by Dr. Raymond Lai^13^ at University of Alberta, Edmonton, AB, Canada and independently confirmed by Sanger sequencing (Genewiz, South Plainfield, NJ). For bacterial expression, the NPM-ALK gene was moved to the pET28a/NPM-ALK construct, transformed into competent cells of Rosetta 2(DE3) (EMD Millipore Corp, MA), a BL21 derivative with added tRNAs designed to enhance the expression of eukaryotic genes containing codons rare in *E. coli*. Inclusion bodies were recovered and solubilized protein was refolded at 25 °C by dropwise addition of refolding buffer under continuous stirring. The refolded protein was first purified in an Ni-NTA Sepharose column. The eluted NPM-ALK protein was further purified using anion exchange chromatography on a Q-Sepharose column pre-equilibrated with 25 mM Tris-HCl, pH 8.0. The protein was eluted in a linear gradient of 0 to 1 M NaCl over 20 column volumes. The purity and molecular mass of the eluted protein were confirmed by SDS-PAGE on a 4–15% Mini-PROTEAN TGX Precast Gel (Bio-Rad) and quantified using the BCA Protein Assay (Thermo Scientific, Rockford, IL) with bovine serum albumin (BSA) as standard. Purified recombinant protein was aliquoted and stored in 50% glycerol at −20 °C for later use. We have deposited the construct in Addgene (pMCRW1; #122462). Additional experimental details for the expression and purification of the recombinant NPM-ALK fusion protein are available in the Supplementary Information.

### Immunodetection and quantification of NPM-ALK fusion protein

Cell lysates (in the non-denaturing Cell Lysis Buffer) were assayed by ELISA for NPM-ALK fusion protein and β-actin. Recombinant NPM-ALK fusion protein was serially diluted in PBS/1% BSA and was used as the calibration standard on the same 96-well microwell plate as the cell lysates in ELISA (experimental details are available in the Supplementary Information). We confirmed the reproducibility of sample preparation and the uniformity of cell numbers used per assay using the PathScan Total β-Actin Sandwich ELISA Antibody Pair #7881 (Cell Signaling Technology; capture: β-actin rabbit monoclonal antibody; detection: pan-actin mouse monoclonal antibody; HRP-linked anti-mouse secondary IgG antibody). β-actin was used to confirm the ELISA results by 2-D electrophoresis and also can be potentially used for later normalization of number of cells in biopsy specimens.

## Supplementary information

Supplementary information.
