# Supplementary material for: Recombinant expression, characterization, and quantification in human cancer cell lines of the Anaplastic Large-Cell Lymphoma-characteristic NPM-ALK fusion protein
Source: Sci Rep. 2020 Mar 19;10:5078. doi: 10.1038/s41598-020-61936-w (PMC7081362; doi:10.1038/s41598-020-61936-w)
Supplement: Supplementary file 1 — Supplementary information. [file 41598_2020_61936_MOESM1_ESM.pdf]

Supplementary Information

Recombinant expression, characterization, and quantification in human cancer cell lines of the Anaplastic Large-Cell Lymphoma-characteristic NPM-ALK fusion protein

Katerina Kourentzi*, Mary Crum, Ujwal Patil, Ana Prebisch, Dimple Chavan, Binh Vu, Zihua Zeng, Dmitri Litvinov, Youli Zu*, and Richard C. Willson*

*Corresponding Authors
E-mail: willson@uh.edu (RCW), edkourentzi@uh.edu (KK) and yzu@houstonmethodist.org (YZ)

Materials. Restriction endonucleases and DNA-modifying enzymes were purchased from New England Biolabs (Ipswich, MA). Oligonucleotides were purchased from Integrated DNA Technologies (Coralville, IA). HRP-conjugated horse anti-mouse IgG antibody (#7076; used at 1000-fold dilution of the stock) was from Cell Signaling Technology (Danvers, MA); HRP-conjugated donkey anti-goat IgG antibody (sc-2020; used at 1000-fold dilution of the stock) was from Santa Cruz Biotechnology, Inc; HRP-conjugated goat anti-rabbit IgG antibody (#32460; used at 500-fold dilution of the stock) was from Thermo Scientific (Rockford, IL); 1-Step™ Ultra TMB-ELISA Substrate Solution (#34028) was from Thermo Scientific. Phosphate-buffered saline (PBS) tablets (T9181), pH 7.4, were from Clontech Laboratories, Inc. (Mountain View, CA). Non-denaturing Cell Lysis Buffer (#9803; 20 mM Tris-HCl, pH 7.5, 150 mM NaCl, 1 mM Na_2EDTA, 1 mM EGTA, 1% Triton, 2.5 mM sodium pyrophosphate, 1 mM β-glycerophosphate, 1 mM Na_3VO_4 1 μg/ml leupeptin), Phenylmethanesulfonyl Fluoride (PMSF, #8553) and Protease Inhibitor Cocktail (#5871) were from Cell Signaling Technology; M-PER Mammalian Protein Extraction Reagent containing the zwitterionic detergent CHAPS[1] in 25 mM Bicine buffer, pH 7.6, was from Thermo Scientific. Bovine serum albumin (BSA, A7906-50G), TWEEN® 20 (Molecular Biology Grade, P9416-100ML), Nunc® MicroWell™ 96 well polystyrene plates (P7366-1CS) and all other reagents and buffers were from Sigma-Aldrich, Inc. (St. Louis, MO).

Production of recombinant fusion protein. The human NPM-ALK gene in the pCDNA3/NPM-ALK construct was kindly provided by Dr. Raymond Lai[2] at University of Alberta, Edmonton, AB, Canada. Sanger sequencing

done by Genewiz (South Plainfield, NJ) independently confirmed the fusion gene sequence using T7 universal PCR primers as well as previously-described PCR primers[3] (for the ALK part: NPM_F: 5'-GGGCCAGTGCATATTAGTGGA or BreakPoint_F: 5'-CTTAGTAGTGTACCGCCGGAAG; for the NPM part of the fusion gene: ALK_R: 5'-TGTACTCAGGGCTCTGCAGCT-3').

We initially explored *in vitro* transcription/ translation of NPM-ALK fusion protein from the mammalian expression vector (pCDNA3). *In vitro* transcription/translation of the construct was performed with the TNT coupled rabbit reticulocyte lysate system with T7 RNA polymerase (Promega, Madison, WI). Expression of the NPM-ALK fusion protein was confirmed with an *in house* sandwich ELISA using antibodies from Thermo Scientific (capture: anti NPM1 antibody (MA5-12508) targeting a fusion peptide containing the N-terminus of NPM fused to 14 amino acids of ALK; detection: anti ALK antibody (MA5-14528) targeting the tyrosine kinase catalytic domain and part of the C-terminus of the NPM-ALK transcript). However, the yield of fusion protein by this method was not adequate for our purposes.

For bacterial expression, the NPM-ALK gene was originally cloned from pCDNA3/NPM-ALK into pET28b at EcoRI (192) and XhoI (158) sites. It was then amplified using the following primers: NPM- F 5'-GAATCATATGGAAGATTCGATGGACATG and the universal primer T7-Terminator 5'-GCTAGTTATTGCTCAGCG. The amplified DNA fragment (2,164 bp) was agarose gel separated, excised, digested with NdeI and XhoI and ligated into pET28a vector between the NdeI (238) and XhoI (158) sites (adding an N-terminal hexahistidine (his_6)-tag to the expressed protein). The pET28a/NPM-ALK construct was transformed into competent cells of Rosetta 2(DE3) (EMD Millipore Corp, MA), a BL21 derivative with added tRNAs designed to enhance the expression of eukaryotic genes containing codons rare in *E. coli.* Bacterial cultures (in 1L LB medium supplemented with 50 µg/mL kanamycin and 25 µg/mL chloramphenicol) were grown in 6-L Erlenmeyer flasks with shaking at 220 rpm at 37 ^{o}C to an OD600 of 4. Protein expression was induced with 0.5 mM isopropyl-β-D-thiogalactopyranoside (IPTG) and the cells were allowed to grow for another 3 h at 37 ^{o}C. Cells were then pelleted by centrifugation at 2,500 xg at 4 ^{o}C for 10 min. Pelleted cells were again resuspended in 30 mL PBS, pelleted again by centrifugation and finally resuspended in 30 mL PBS. The resuspended cells were disrupted using 5 freeze/thaw cycles followed by passage through a French Pressure Cell Press (Thermo Spectronic) at 10,000 psi. Inclusion bodies were recovered by centrifugation at 16,000 xg at 4 ^{o}C for 25 min. Each *ca.* 85 mg pellet containing inclusion bodies was resuspended in 1 mL wash buffer (50 mM Tris-HCl, pH 8.0, 2 M urea and 0.5% Triton X-100) by intermittent vortexing for 30 sec. The washed inclusion bodies were pelleted by centrifugation at 16,000 xg at 4 ^{o}C for 25 min, and the washed pellet resuspended in 1 mL 50 mM Tris-HCl, pH 8.0, followed by centrifugation at 16,000 xg at 4°C for 25 min. The centrifuged pellet was then mixed with 850 µL solubilization buffer (50 mM Tris-HCl, pH 8.0, 7.5 M urea, 500 mM NaCl, 400 mM L-arginine, 10 mM DTT) on a rotator for 120 min at 25 ^{o}C. The solubilized protein was refolded at 25 ^{o}C by dropwise addition at 5

μL/min to 18 mL of refolding buffer (50 mM Tris-HCl, pH 8.0, 500 mM NaCl, 5% (v/v) glycerol, 5% (w/v) sucrose and 400 mM L-arginine) under continuous stirring. The refolding mixture was filtered through a 0.45 μm filter and loaded onto a Ni-NTA Sepharose column (42 mm H x 5 mm ID) previously equilibrated with 50 mM Tris-HCl, pH 8.0 and 500 mM NaCl at a flow rate of 0.4 mL/min. The protein was eluted between 125-210 mM imidazole in a gradient of 0 to 500 mM imidazole in 50 mM Tris-HCl, pH 8.0 and 500 mM NaCl over 20 column volumes. The NPM-ALK fusion protein was further purified using anion exchange chromatography on a Q-Sepharose column (42 mm H x 5 mm ID) pre-equilibrated with 25 mM Tris-HCl, pH 8.0. The protein was eluted in a linear gradient of 0 to 1 M NaCl over 20 column volumes. The purity and molecular mass of the eluted protein were confirmed by SDS-PAGE on a 4-15% Mini-PROTEAN TGX Precast Gel (Bio-Rad). The BCA Protein Assay (Thermo Scientific, Rockford, IL) was used for quantitation of the eluted protein with bovine serum albumin (BSA) as standard. Purified recombinant protein was aliquoted and stored in 50% glycerol at -20 °C for later use.

Mass Spectrometry. The recombinant fusion protein was characterized at the Proteomics Facility, U.T. MD Anderson Cancer Center, Houston, TX. Purified recombinant NPM-ALK fusion protein was acetone precipitated (5:1) overnight at -20 °C and digested with 200 ng modified trypsin (sequencing grade, Promega, Madison WI) in the presence of RapiGest (Waters, Milford MA) for 18 h at 37 °C. Resulting peptides were analyzed by high-sensitivity LC-MS/MS on an Orbitrap-Fusion mass spectrometer (Thermo Scientific, Waltham MA). Proteins were identified by database searching of the fragment spectra against the SwissProt (EBI) protein database using Mascot (v 2.6.2, Matrix Science, London, UK) through Proteome Discoverer (v 1.4, Thermo Scientific). Database search settings were: mass tolerances, 10 ppm precursor, 0.8d fragments; variable modifications, methionine sulfoxide, pyro-glutamate formation; Trypsin, up to 2 missed cleavages. FDR estimates were from Proteome Discoverer. All the identified peptides were above the MASCOT threshold score for identity with a p-value of <0.05. The analysis identified a total of 37 unique peptides (28 for full length ALK and 9 for full length NPM), with peptides ranging in length from 9 to 36 amino acid residues.

Cell lines. Karpas 299 (CD30+) and SU-DHL-1[4] human ALCL cell lines were obtained from Dr. Mark Raffeld at the National Cancer Institute/National Institutes of Health. Jurkat T-cell, CA46 B-cell and U937 histiocytic human lymphoma cell lines negative for both full-length wild type ALK and NPM-ALK fusion proteins were purchased from American Type Culture Collection (ATCC; Manassas, VA). IMR-32 human neuroblastoma cell line, negative for the NPM-ALK fusion protein but positive for full-length wild type ALK protein[5-7], was also purchased from American Type Culture Collection (ATCC; Manassas, VA). All cells were grown in RPMI 1640 medium supplemented with 10% fetal bovine serum, 100 unit /mL penicillin and 100 µg/mL streptomycin at 37 °C in a humidified atmosphere with 5% CO_2. After two days' culture, cells were harvested and viable cells were counted using trypan blue stain. After washing twice in PBS, cells were centrifuged, resuspended in RPMI 1640 medium with 10% DMSO and stored at -80 °C (1.5 to 5 x10^6 cells/per vial) for later use.
Frozen cells were thawed in a pre-warmed water bath at 37 °C for 10 min, transferred to clean Eppendorf tubes and centrifuged at 500 xg for 10 min at 4 °C. Cell pellets were washed in 1 mL PBS, and centrifuged at 500 xg for 10 min at 4 °C. After removal of the supernatant, cells were resuspended in the non-denaturing cell lysis buffer (#9803; Cell Signaling Technology, Inc.; chosen after screening of 3 lysis reagents; see Figure 2, main text) and incubated on ice for 5 min according to the manufacturer's instructions. Thereafter, the lysates were centrifuged at 14,000 xg for 10 min at 4 °C to remove cell debris, the supernatant transferred to new vials and total protein quantified by BCA assay with bovine serum albumin (BSA) as a standard.

Optimized ELISA. The wells of a Nunc® MicroWell™ 96 well polystyrene plate were coated with 100 µL of 2.5 µg/mL anti-NPM-ALK capture antibody (ab180607; Abcam) diluted in PBS, pH 7.5 and the plate was incubated at 4 °C overnight. The antibody solution was removed and the wells were passivated with 300 µL PBS/3% BSA for 1 h at 37 °C. The wells were then washed three times with PBS/0.1% TWEEN® 20. Samples (100 µL) were then added to each well, and the plate was incubated for 1.5 h at 37 °C. Then the wells were emptied and washed three times with PBS/0.1% TWEEN® 20. Detection antibodies (#3791; anti-ALK mouse monoclonal antibody; Cell Signaling Technologies; 100 µL, 0.5 µg/mL in PBS/0.1% BSA) were added to each well, and the plate was incubated for 30 min at 37 °C. Supernatant was removed and the wells were washed three times with PBS/0.1% TWEEN® 20. Anti-mouse IgG horseradish peroxidase conjugates (100 µL/well; used at 1000-fold dilution of the stock in PBS/0.1% BSA) were added and the plate was incubated for 30 min at 37 °C. Supernatant was removed, the wells were washed three times with PBS/0.1% TWEEN® 20 and 100 µL 1-Step™ Ultra TMB-ELISA Substrate Solution was added to the wells. Following incubation for 20 min at 37 °C, 50 µL 2 M sulfuric acid was added to each well, and the absorbance at 450 nm was measured with an Infinite® M200 PRO multimode reader. All washing steps were performed on a Tecan HydroFlex microplate washer using a

typical wash program (3 cycles, Normal Mode, Aspirate Time 1 sec, Aspirate rate 2, Head Speed 10 mm/sec; z-position: overflow, Dispense Volume 400 μL/well, Dispense Rate 300 μL/sec).

Cellular protein analysis by two-dimensional gel electrophoresis. Cultured Karpas 299 cells ($5x10^6$ cells/sample) were lysed in 200 μL of sample buffer containing 7 mM urea, 2 mM thiourea, 4% CHAPS, 10 mM Tris, pH 8.8, and 5 mM magnesium acetate. Cellular protein concentrations were determined using a Bradford assay kit (BioRad, Hercules, CA, USA) and diluted with sample buffer to yield a final protein concentration of 5 mg/mL. For protein labeling, 400 pmol of Cy3 NHS ester was added to 10 μL of sample containing 50 μg protein and incubated for 30 min on ice in the dark. The labeling reaction was terminated by adding 1 mM lysine and incubating for 10 min. The labeled cellular protein samples were then made up to a final volume of 450 μL in the IEF sample buffer containing 0.5% ampholytes, 0.1% bromophenol blue, and 12 μL/mL DeStreak reagent (GE Healthcare).

For the first-dimension electrophoresis, isoelectric focusing was carried out using the GE Healthcare IPGphor II pH 3–10 nonlinear, 24-cm immobilized pH gradient (IPG) strips (GE Healthcare) overnight. Subsequently, the IPG strips were loaded onto 8-18% SDS-PAGE gels (24x20 cm) for the second-dimension electrophoresis. Post-electrophoresis, the gels were scanned in a Typhoon 9400 laser scanner (GE Healthcare) and digital images were recorded using ImageQuant (GE Healthcare) software. Individual protein spots observed in the image were then quantified and compared after volumetric rendering using the differential in-gel analysis (DIA) algorithm within DeCyder (v5.5; GE Healthcare) software. Finally, the spot of NPM-ALK protein on the second-dimension gel was cut out and peptide sequencing was performed in the Translational Proteomics Core at UTHealth, essentially as previously described[8]. Protein identification was confirmed by a MASCOT protein score of 102 and protein score confidence interval (C.I. %) of 100%.

Mixing experiments. Mixing experiments were performed to estimate the sensitivity of the NPM-ALK ELISA to detect ALK-positive ALCL cells in whole cell lysates. Karpas 299 cells were serially diluted in 2 million ALK-negative U937 cells and whole cell extracts were prepared in Cell Lysis Buffer. Immunodetection of cellular NPM-ALK fusion protein in the whole cell extracts was performed as described in the optimized ELISA section above. We observed a sensitivity of $5x10^{-3}$ (0.5% Karpas 299 ALCL cells).

REFERENCES

1 Yang-Boja, E., DeFilippes, F. & Fales, H. M. Electrospray mass spectra of three proprietary detergents. *Anal. Biochem.* **285**, 205-210, doi:10.1006/abio.2000.4734 (2000).

2 Wu, F., Wang, P., Young, L. C., Lai, R. & Li, L. Proteome-wide identification of novel binding partners to the oncogenic fusion gene protein, NPM-ALK, using tandem affinity purification and mass spectrometry. *Am. J. Pathol.* **174**, 361-370, doi:10.2353/ajpath.2009.080521 (2009).

3 Maes, B. *et al.* The NPM-ALK and the ATIC-ALK fusion genes can be detected in non-neoplastic cells. *Am. J. Pathol.* **158**, 2185-2193, doi:10.1016/S0002-9440(10)64690-1 (2001).

4 Turturro, F., Frist, A. Y., Arnold, M. D., Seth, P. & Pulford, K. Biochemical differences between SUDHL-1 and KARPAS 299 cells derived from t(2;5)-positive anaplastic large cell lymphoma are responsible for the different sensitivity to the antiproliferative effect of p27Kip1. *Oncogene* **20**, 4466, doi:10.1038/sj.onc.1204582 (2001).

5 Chaudhari, N., Talwar, P., Lefebvre D'hellencourt, C. & Ravanan, P. CDDO and ATRA Instigate Differentiation of IMR32 Human Neuroblastoma Cells. *Front Mol Neurosci* **10**, 310, doi:10.3389/fnmol.2017.00310 (2017).

6 Dirks, W. G. *et al.* Expression and functional analysis of the anaplastic lymphoma kinase (ALK) gene in tumor cell lines. *Int J Cancer* **100**, 49-56, doi:10.1002/ijc.10435 (2002).

7 Lamant, L. *et al.* Expression of the ALK tyrosine kinase gene in neuroblastoma. *Am J Pathol* **156**, 1711-1721, doi:10.1016/S0002-9440(10)65042-0 (2000).

8 Lou, B., Engler, D., Dubinsky, W., Wu, J. & Vigneswaran, N. Acquiring Metastatic Competence by Oral Squamous Cell Carcinoma Cells Is Associated with Differential Expression of alpha-Tubulin Isoforms. *J Oncol* **2012**, 491685, doi:10.1155/2012/491685 (2012).

9 Elenitoba-Johnson, K. S. J. *et al.* Proteomic identification of oncogenic chromosomal translocation partners encoding chimeric anaplastic lymphoma kinase fusion proteins. *P Natl Acad Sci USA* **103**, 7402-7407, doi:10.1073/pnas.0506414103 (2006).

SI FIGURES

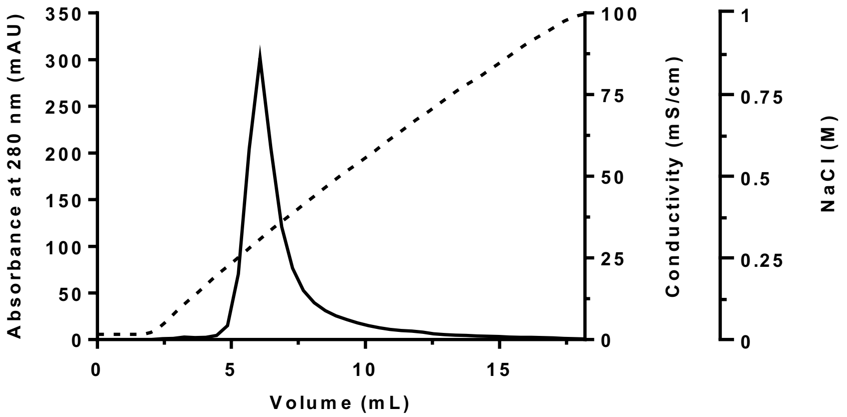

Figure S1. Chromatogram of recombinant NPM-ALK purification using anion exchange chromatography with gradient elution. The affinity purified NPM-ALK fraction was loaded on a 42 mm H x 5 mm ID Q-Sepharose column, and elution was by an increasing NaCl gradient (0-1 M over 20 column volumes, i.e. 16 mL) in 25 mM Tris-HCl, pH 8.0. The NPM-ALK fusion protein eluted at 350 mM NaCl. Two 1-ml fractions were pooled; at 5-6 and 6-7 mL elution volume.

MEDSMDMDMSPLRPQNYLFGCELK**ADKDYHFKVDNDENEHQLSLRTVSLGAGAKDELHIVEAEA**
MNYEGSPIKVTLATLKMSVQPTVSLGGFEITPPVVLRLKCGSGPVHISGQHLVVYRR**KHQELQAM**
QMELQSPEYKLSKLR**TSTIMTDYNPNYCFAGKTSSISDLKEVPRKNITLIRGLGHGAFGEVYEGQ**
VSGMPNDPSPLQVAVKTLPEVCSEQDELDFLMEALIISK**FNHQNIVRCIGVSLQSLPRFILLELMAG**
GDLKSFLRETRPRPSQPSSLAMLDLLHVARDIACGCQYLEENHFIHRDIAARNCLLTCPGPGRVAK**I**
GDFGMARDIYRASYYR**KGGCAMLPVKWMPPEAFMEGIFTSKT**DTWSFGVLLWEIFSLGYMPYPSK
SNQEVLEFVTSGGRMDPPKNCPGPVYRIMTQCWQHQPEDRPNFAIILERIEYCTQDPDVINTALPIEY
GPLVEEEEKVPVRPK**DPEGVPPLLVSQQAKREEERSPAAPPPLPTTSSGKA**AKKPTAAEVSVRVPR**G**
PAVEGGHVNMAFSQSNPPSELHKVHGSR**NKPTSLWNPTYGSWFTEKPTKK**NNPIAKKEPHDRGNL
GLEGSCTVPPNVATGR**LPGASLLLEPSSLTANMKEVPLFRL**RHFPCGNVNYGYQQQGLPLEAATAPG
AGHYEDTILK**SKNSMNQPGP**

Figure S2. Mapping of recombinant NPM-ALK protein by LC-MS/MS. Purified recombinant NPM-ALK fusion protein was trypsin-digested and peptide sequencing was performed by LC-MS/MS at the Proteomics Facility, U.T. MD Anderson Cancer Center (Houston, TX). Detected peptide masses were identified by MASCOT against sequences in the SwissProt database (2016_07; 551705 sequences; 197114987 residues). The identified peptides matching to the GenBank sequence of human NPM-ALK fusion protein (#AAA58698.1) are shown in red. The underlined sequence denotes the NPM portion of the fusion protein. Sequence coverage was calculated to be 59.4% (404 amino acids were identified out of the 680 amino acids of the fusion protein); previously reported MS/MS mapping of trypsin-digested NPM-ALK protein in SUDHL-1 cells yielded 29.9% coverage including the fusion junction[9].

Capture antibody \ Detection antibody	ab3333	ab180607	ab14528	ab24412
ab3791	1.04	1.0	0.64	0.11
ab12508	0.16	0.15	0.18	0.13

Detection antibody \ Capture antibody	ab3333	ab180607	ab14528	ab24412
ab3791	3.03	1.16	0.29	0.11
ab12508	0.34	0.53	N/A	0.18

Figure S3. ELISA-based antibody screening results with lysates from 5,000 Karpas 299 cells (NPM-ALK-positive) or 20,000 Jurkat cells (NPM-ALK-negative). Green color denotes maximum ELISA OD signal at 450 nm, red lowest, with a smooth color gradient in between. The following antibodies were tested: anti-ALK ab3791 (CTC, #3791 mouse monoclonal IgG targeting an ALK C-terminus fragment), anti-ALK ab3333 (CTC, #3333, rabbit monoclonal IgG1 targeting a recombinant peptide surrounding aa 1475 of ALK), anti-fusion ab180607 (abcam, ab180607 rabbit monoclonal IgG targeting a proprietary NPM1/ALK peptide), anti-ALK ab14528 (Thermo, #MA5-14528 rabbit monoclonal IgG targeting a recombinant protein corresponding to a region, which spans the tyrosine kinase catalytic domain and part of the C-terminus of the NPM-ALK transcript), anti-NPM ab24412 (abcam, #ab24412 rabbit polyclonal IgG targeting the N-terminus aa 6-55 of NPM), and anti-fusion ab12508 (Thermo, #MA5-12508 mouse monoclonal IgG targeting a GST fusion protein containing the N-terminus part of NPM fused to 14 aa of ALK). A secondary anti-species HRP-conjugated antibody (HRP-conjugated horse anti-mouse IgG antibody (#7076) from Cell Signaling Technology or HRP-conjugated goat anti-rabbit IgG antibody (#32460) from Thermo) along with 1-Step™ Ultra TMB-ELISA Substrate Solution was used for signal development. All antibody combinations tested gave <0.1 OD signal with the negative control Jurkat cells and the optimized lysis sample preparation protocol.

N/A: anti-NPM antibody ab14528 was not tested as a capture antibody since the endogenous NPM protein will compete with the NPM-ALK fusion protein for binding to this antibody.

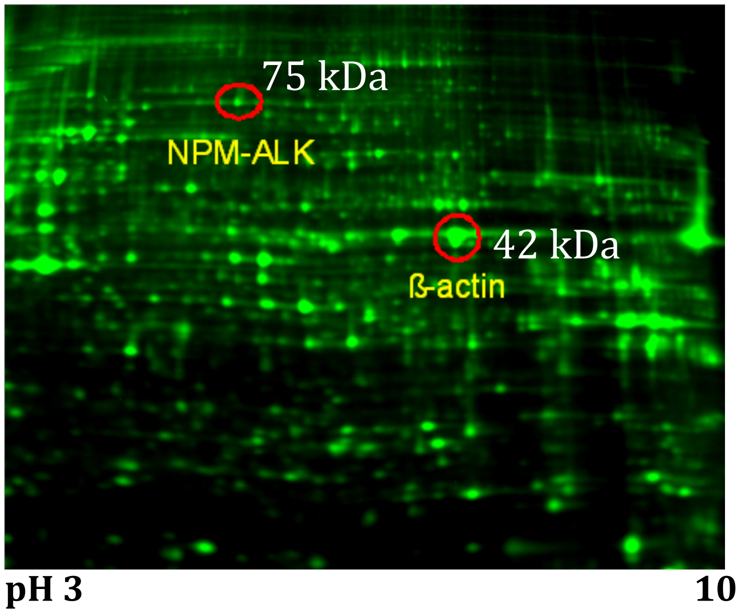

Figure S4. **2-D gel electrophoretic measurement of NPM-ALK fusion protein expression in ALCL cells.** Cultured Karpas 299 cells were lysed, and total cellular proteins were labeled with Cy3 fluorescent dye and then separated by two-dimensional gel electrophoresis as described in Methods. For identification, individual proteins/spots were collected and sequenced by LC-MS. Cellular NPM-ALK protein and β-actin, one of the major cellular proteins, are indicated. The gel was scanned in a Typhoon 9400 laser scanner and digital images were recorded using ImageQuant software. Individual protein spots observed in the image were then quantified and compared after volumetric rendering using the differential in-gel analysis (DIA) algorithm within DeCyder (v5.5; GE Healthcare). The relative molar ratio of β-actin to NPM-ALK protein was calculated approximately at 80-100:1.

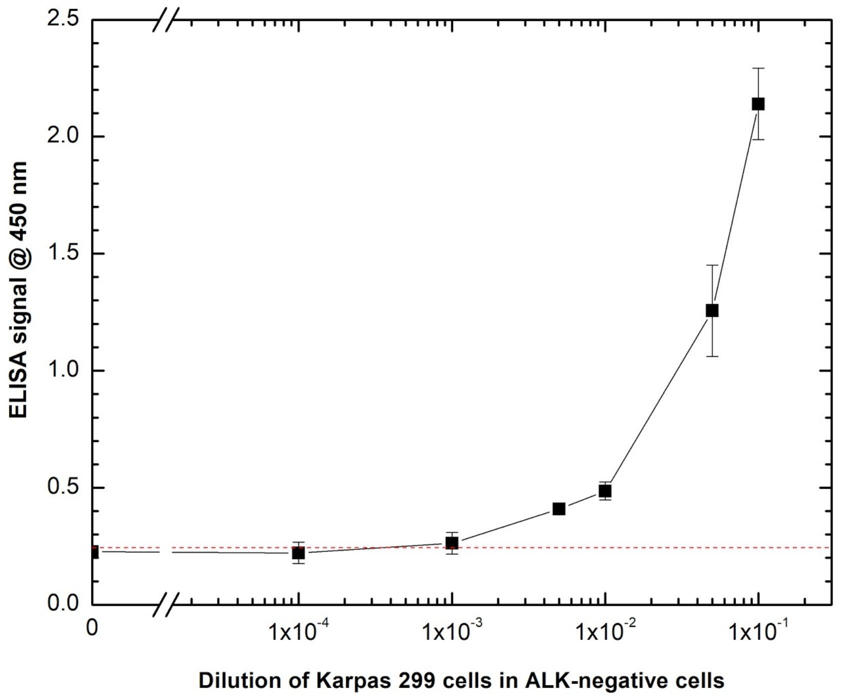

Figure S5. Detection of Karpas 299 ALCL cells by the NPM-ALK ELISA in "normal" ALK- negative U937 cells. ALK-positive Karpas 299 cells were serially diluted in 2 million ALK-negative U937 cells and whole cell extracts were prepared in Cell Lysis Buffer. Immunodetection of cellular NPM-ALK protein in whole cell extracts was performed by ELISA with the ab180607 (capture)/#3791 (detection) antibody pair. An HRP-conjugated horse anti-mouse IgG antibody (#7076) from Cell Signaling Technology along with 1-Step™ Ultra TMB-ELISA Substrate Solution was used for signal development (n=3, average ± 1 SD). The red dotted line signifies the detection limit cutoff taken as the mean plus three times the standard deviation ($\mu \pm 3\sigma$) of the ELISA signal of the negative sample containing no Karpas 299 cells.
